# The Transmural Trauma Care Model (TTCM) for the rehabilitation of trauma patients is effective in improving patient related outcome measures: a non-randomized controlled trial

**DOI:** 10.1186/s12913-019-4547-6

**Published:** 2019-11-08

**Authors:** Suzanne H. Wiertsema, Johanna M. van Dongen, Edwin Geleijn, Heleen Beckerman, Frank W. Bloemers, Raymond W. J. G. Ostelo, Vincent de Groot

**Affiliations:** 1 Department of Rehabilitation Medicine, Amsterdam UMC, Vrije Universiteit Amsterdam, Amsterdam Public Health research institute, De Boelelaan 1118, 1081 HZ Amsterdam, Netherlands; 2 Department of Health Sciences, Vrije Universiteit Amsterdam, Amsterdam Public Health research institute, Amsterdam, Netherlands; 3 Department of Rehabilitation Medicine, Amsterdam UMC, Vrije Universiteit Amsterdam, Amsterdam, Netherlands; 4 Department of Trauma Surgery, Amsterdam UMC, Vrije Universiteit Amsterdam, Amsterdam, Netherlands; 5 Department of Epidemiology and Biostatistics, Department of Health Science, Amsterdam UMC, Vrije Universiteit Amsterdam, Amsterdam Public Health research institute, Amsterdam, Netherlands

**Keywords:** Trauma, Fractures, Rehabilitation, HR-QOL, Functional outcome, Transmural care, Organization

## Abstract

**Background:**

The Transmural Trauma Care Model (TTCM) is a refined post-clinical rehabilitation approach, in which a multidisciplinary hospital-based team guides a network of primary care physical therapists in the treatment of trauma patients. The objective of this study was to assess the effectiveness of the TTCM compared to regular care.

**Methods:**

A controlled-before-and-after study was performed in a level 1 trauma center. The TTCM includes four elements: 1) a multidisciplinary team at the outpatient clinic, 2) coordination and individual goal setting for each patient by this team, 3) a network of primary care physical therapists, 4) E-health support for transmural communication. Intervention group patients were prospectively followed (3, 6 and 9 months). The control group consisted of 4 clusters of patients who either had their first consultation at the outpatient clinic 0, 3, 6 or 9 months ago. Outcomes included generic- and disease-specific health-related quality of life (HR-QOL), pain, functional status, patient satisfaction, and perceived recovery. Between-group comparisons were made using linear regression analyses. The recovery pattern of intervention group patients was identified using longitudinal data analysis methods.

**Results:**

A total of 83 participants were included in the intervention group. In the control group, 202 participants were included (68 in the baseline cluster, 26 in the 3-month cluster, 51 in the 6-month cluster, 57 in the 9-month cluster). Between-group differences were statistically significant in favor of the intervention group for disease-specific HR-QOL at 9 months, pain at 6 and 9 months, functional status at 6 and 9 months, patient satisfaction at 3, 6 and 9 months, and perceived recovery at 6 months. No significant differences were found between groups for generic HR-QOL at any time point. Generic HR-QOL, disease-specific HR-QOL, pain, and functional status significantly improved in a linear fashion among intervention group patients during the nine-month follow-up period.

**Conclusions:**

This study provides preliminary evidence that the TTCM is effective in improving patient related outcome measures, such as disease-specific HR-QOL, pain and functional status. A multicenter, and ideally randomized controlled trial, is required to confirm these results.

**Trial registration:**

The trial is registered at the Dutch Trial Register (NTR5474). Registered 12 October 2015. Retrospectively registered.

## Background

Traumatic injury-related mortality accounts for almost 10% of the global annual mortality. Moreover, major trauma accounts for the highest mortality rate among people under 40 years of age, compared to any other disease [1, 2]. As a consequence, traumatic injury is responsible for the highest loss of Disability-Adjusted Life Years (DALYs) worldwide. Each year, trauma costs the global population about 300 million years of healthy life, equaling 11% of DALYs lost [3]. Furthermore, in adults younger than 45 years, major trauma is the most important cause of long-term functional limitations [4].

Many trauma patients have more than one fracture. Fractures, and those of the lower extremities in particular, significantly impact a patient’s functional status and health-related quality of life (HR-QOL) [5, 6]. On top of that, the economic burden of trauma to society is extensive, for example, the societal cost of an operatively treated vertebral fracture was estimated at €66.000 per patient [7]. Furthermore, Fakhry et al. showed that trauma patients represent a significant and increasing institutional cost, of which ICU costs per trauma patient were the largest single category [8]. During the last decades, a significant decrease in mortality has been achieved among severe trauma patients through the optimization of *pre-hospital* and *in-hospital* trauma care [9–12]. As further reductions in mortality rates are therefore expected to be trivial, the focus of trauma care has shifted from aiming to reduce mortality rates to aiming to improve trauma patients’ HR-QOL and outcome [9–12]. As a consequence, HR-QOL has become one of the most important outcome measures in studies among severely injured trauma patients [13, 14], whereas relatively few studies have focused on measuring HR-QOL amongst mildly to moderately injured patients [15, 16].

To further improve outcome and HR-QOL among mild, moderate, and severe trauma patients, increased attention is required for optimizing the rehabilitation process after in-hospital trauma care [17–19]. Research among other patient groups indicates that an improved organization of the post-clinical rehabilitation process can lead to better outcomes [20–22]. For example, a study in patients with Parkinson’s disease indicates that a post-clinical care model in which rehabilitation is organized in a network of experienced and specialized healthcare providers results in better clinical outcomes and lower costs compared to regular care models [20]. Furthermore, a feasibility study among patients with hip or knee osteoarthritis found a care model, in which primary care providers were guided by a clinical case manager, to significantly improve patients’ outcome and HR-QOL [21].

Given the above, we developed a new Transmural Trauma Care Model (TTCM) for trauma patients. The core of the TTCM is a continuous feedback loop, in which a multidisciplinary hospital-based team supervises a network of primary care physical therapists.

The aim of the current study is to assess the following research questions:

1. What is the effectiveness of the TTCM on HR-QOL (generic- and disease-specific), pain, functional status, patient satisfaction and perceived recovery, compared to regular care, in trauma patients with at least one fracture?
2. What is the recovery pattern of trauma patients receiving the TTCM, during the 9 month follow-up period, regarding HR-QOL (generic- and disease-specific), pain and functional status?

## Methods

The study protocol of the current study, with detailed descriptions of its design and methods, has been published elsewhere [23]. Alongside the present study (assessing the effectiveness of the TTCM), the *cost-effectiveness* of the TTCM was evaluated in an economic evaluation, of which the results were recently published [24]. Consequently, some parts of the method section below are overlapping with the aforementioned publications (i.e. patients, inclusion procedure, intervention- and control conditions and outcome measures). An abridged version of the earlier published study protocol, is presented below.

### Design

A modified controlled-before-and-after study was conducted at the outpatient clinic for trauma patients of the Amsterdam UMC, Vrije Universiteit Amsterdam (VUmc), the Netherlands [23, 24]. In a true controlled-before-and-after study both study groups are prospectively followed [25]. However, in the present study, only the intervention group was prospectively followed, while control group data were collected cross-sectionally.

From January to March 2014, control group data were collected among patients who received regular care. The control group consisted of 4 clusters of patients. The baseline, 3-month, 6-month, and 9-month clusters contained patients who had their first consultation at the outpatient clinic within 1 week ago, or 3 months ago, 6 months ago, and 9 months ago, respectively. All control group patients were only measured once at the time point that corresponds to the cluster they belong to.

**Fig. 1:**
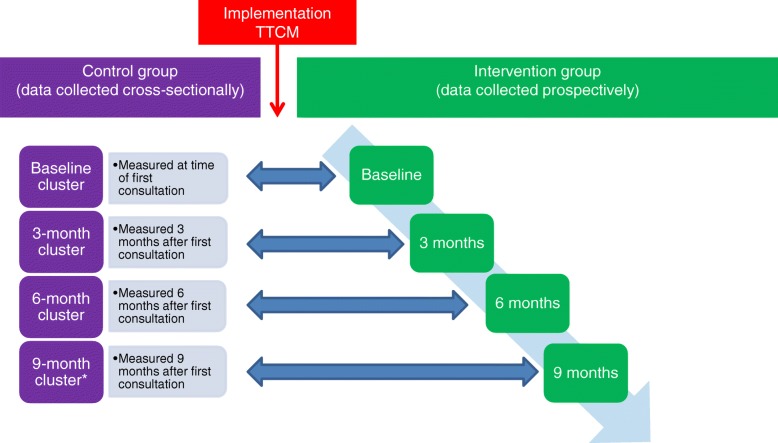
Study design

From April to May 2014, the TTCM was implemented. Subsequently, intervention group participants were recruited from June 2014 to April 2015, after which they received care according to the TTCM. All intervention group patients were prospectively followed for 9 months with measurements at baseline and 3, 6 and 9 months after their first consultation at the outpatient clinic. A graphical representation of the study design can be found in Fig. 1.

The medical ethics committee of the VUmc assessed the present study, and decided that the Dutch Medical Research Involving Human Subjects Act (WMO) was not applicable (registered under number 2013.454). All participants gave informed consent. The trial is registered at the Dutch Trial Register (NTR5474) and adheres to the CONSORT guideline.

### Patients

Operatively and non-operatively treated trauma patients were included, regardless of whether or not they were admitted to the hospital. Eligible patients had to have at least one traumatic fracture (i.e. upper and lower extremity fractures, spinal fractures, hip fractures), had to be 18 years or older, had to rehabilitate in primary care and had to be able to fill out Dutch online questionnaires. Patients were excluded if they had non-traumatic (pathological) fractures, or traumatic brain injury, or cognitive limitations. Furthermore patients were excluded if their rehabilitation occurred in a clinical tertiary care setting, or if they lived more than 30 km away from the Amsterdam UMC, VUmc.

The recruitment procedure of potential participants took place as earlier described in the study protocol [23, 24]. Control group participants were selected from the central trauma registry of the trauma region “North West Netherlands”. All sequential patients were contacted by telephone by one of the investigators and received information about the study’s purpose and procedures. In- and exclusion criteria were verified by the principle investigator, after which patients were allocated to their respective cluster. Eligible patients who were willing and able to participate received an email inclosing a link to an online questionnaire. Clicking the link to the online questionnaire served as informed consent. A reminder email was send after 1 week and again after another week of non-responding. In case of patients not replying to both emails, one of the coordinating investigators contacted the patient by telephone.

Intervention group participants were identified during their first consultation at the outpatient clinic as described in the study protocol [23, 24]. Potentially eligible patients were informed about the study’s purpose and procedures by one of the investigators and in- and exclusion criteria were verified. Eligible patients who were willing and able to participate received an email inclosing a link to the first online questionnaire. Clicking the link to the online questionnaire served as informed consent. A reminder email was send after 1 week and, if necessary, again after another week of non-responding. One of the coordinating investigators contacted the patient by telephone, in case of patient’s not replying to both reminder emails. Then, patients were prospectively followed, with measurements at 3, 6, and 9 follow-up.

### Intervention conditions

Pre- and in- hospital trauma care was similar for both study groups, the intervention phase started at the outpatient clinic for trauma patients.

#### The transmural trauma care model (TTCM)

Patients in the intervention group received care according to the TTCM. A detailed description of the TTCM can be found elsewhere [23, 24]. In brief, the TTCM consists of four main components:

1. *A multidisciplinary team at the outpatient clinic for trauma patients.* The team consists of a trauma surgeon and a trauma-specialized hospital-based physical therapist. The trauma surgeon evaluated the bone- and wound healing process. The physical therapist assessed physical function.
2. *Coordination and individual goal setting for each patient by the multidisciplinary team.* The hospital-based team coordinated the patients’ rehabilitation process in primary care by repeatedly defining individual goals in close cooperation with the patient. To supplement this process, 10 rehabilitation protocols were developed for the most common fractures (e.g. hip fractures, tibial plateau fractures). These protocols were customized for each individual patient by the hospital-based physical therapist, who acted as case manager throughout the rehabilitation process.
3. *An educated and trained network of 40 specialized primary care physical therapists.* This newly developed “VUmc trauma rehabilitation network” consisted of 40 specially trained, physical therapists, all of whom worked in a primary care private practice in the region of Amsterdam [26]. Patients in the intervention group were referred to one of these specialized trauma physical therapists.
4. *Secure email traffic between the hospital-based physical therapist and the primary care physical therapist during the entire rehabilitation process.* A secured email system, developed for healthcare professionals, was connected to both the electronic patient records of the hospital-based physical therapist and the primary care physical therapist.

#### Regular care

Patients in the control group received regular care, during which the trauma surgeon acted as the chief consultant. The trauma surgeon performed consultations at the outpatient clinic for trauma patients, and acted independent of other health care professionals. Based on the clinical judgment of the trauma surgeon, a patient could be referred to a primary care physical therapist, but there was no standardized policy for referral of control group patients. Throughout the patients’ rehabilitation in primary care, there was hardly any contact between trauma surgeon and primary care physical therapists.

### Outcome assessment

An overview of all outcome measurements is provided in Table 1. Extensive details of the outcome measures can be found elsewhere [23, 24].

**Table 1:** Overview of all outcome measurements

Outcomes	Measurement Instrument	Abbreviation	Items	Itemscore	Interpretation
Primary outcome					
Generic HR-QOL	EQ-5D-3L	EQ-5D-3L	5	1–3	higher utility score: better health
Secondary outcomes					
Disease-specific HR-QOL (upper extremity)	Quick Dash score	Q-DASH	11	1–5	sumscore 0–100, higher score: less function
Disease-specific HR-QOL (lower extremity)	Lower Extremity Functional Scale	LEFS	20	0–4	sumscore 0–80, higher score: better function
Disease-specific HR-QOL (vertebral fractures)	Roland Morris Disability Score	RMDS	24	yes/no	sumscore 0–24, higher score: more disability
Disease-specific HR-QOL (multi trauma patients)	Groningen Activity Restriction Scale	GARS	18	1–4	sumscore 18–72, higher score: more restrictions
Disease-specific HR-QOL (over-all)	Q-DASH, LEFS, RMDS, GARS	DSQOL-OA			sumscore 0–100, higher score: less function
Pain	Numeric Pain Rating Scale	NPRS	1	0–10	higher score: more pain
Functional status	Patient Specific Function Scale	PSFS	3	100 mm VAS	higher score: less function
Patient satisfaction	Numeric Rating Scale	NRS	3	0–10	higher score: more satisfaction
Perceived recovery	Global Perceived Effect	GPE	1	1–7	higher score: less recovery

#### Baseline characteristics

At baseline, various relevant demographic and trauma-related characteristics were measured (e.g. gender, age, medical history, ISS, the number of days between trauma and first outpatient consultation [TTO]). Baseline characteristics were collected using online questionnaires, supplemented by data derived from electronic patient records.

#### Primary outcome measure

The primary outcome measure was generic HR-QOL, assessed using the EQ-5D-3L [27]. The EQ-5D-3L consists of 5 questions covering 5 health dimensions (i.e. mobility, self-care, usual activities, pain/discomfort and anxiety/depression), all of which contain 3 severity levels. Using the Dutch tariff, the participants’ EQ-5D-3L health states were converted into a utility score, anchored at 0 (dead) and 1 (optimal health).

#### Secondary outcome measures

Secondary outcome measures were disease-specific HR-QOL, pain, functional status, patient satisfaction and perceived recovery.

Depending on the patients’ specific injury type, disease-specific HR-QOL was measured using one of the following disease-specific function scales:

- The Quick Dash for patients with upper extremity fractures, consisting of 11 items, measuring physical function and symptoms on a five-point scale. The overall score ranges from 0 to 100 [28, 29].
- The Lower Extremity Functional Scale (LEFS) for patients with hip fractures or other lower extremity fractures. The LEFS is a 20-item questionnaire with 5 answering options. The overall score ranges from 0 to 80 [30, 31].
- The Roland Morris Disability Score (RMDS) for patients with vertebral fractures. The RMDS is a 24-item questionnaire with 2 answering categories (yes/no). The overall score ranges from 0 to 24 [32, 33].
- The Groningen Activity Restriction Scale (GARS) for multi trauma patients. The GARS is an 18-item questionnaire with 4 response categories, measuring daily activities. The overall score ranges from 18 to 72 [34, 35].

An overall disease-specific HR-QOL score (DSQOL-OA) was calculated by converting the total scores of the aforementioned questionnaires to a scale from 0 to 100. Higher scores indicated that patients experienced more functional problems [24].

The Numeric Pain Rating Scale (NPRS) was used to measure pain. The NPRS is an 11-point scale ranging from 0 (no pain) to 10 (worst possible pain) [36].

Functional status was measured using the Patient Specific Function Scale (PSFS) [37, 38]. Patients identified 3 important activities that they were having difficulty with. Per activity, they were asked to rate their present level of difficulty associated with each activity on a 0–100 mm Visual Analogue Scale (VAS) ranging from 0 (“able to perform activity at same level as before injury or problem”) to 100 (“unable to perform activity”). The activity that was first mentioned by the participants, was used for statistical analysis.

Patient satisfaction was examined on an 11-point Numeric Rating Scale (NRS) ranging from 0 (very dissatisfied) to 10 (excellent). Three patient satisfaction components related to the TTCM were evaluated: 1) the over-all treatment, 2) treatment located at the outpatient clinic, 3) collaboration between the multidisciplinary team at the outpatient clinic and the primary care physical therapist.

Perceived recovery was examined using the Global Perceived Effect (GPE) scale. The GPE quantifies a patient’s subjective improvement on a 7-item scale, ranging from “worse than ever” (1) to “completely recovered” (7) [39]. Success of treatment was achieved when a patient reported 6 or 7 points meaning respectively “much improved” or “completely recovered”.

### Data analysis

The current data analysis section is highly comparable to the version previously described in the study protocol [23].

#### Descriptive statistics

Descriptive statistics were used to compare baseline characteristics between study groups.

#### Handling missing data

Missing data were imputed using Multiple Imputation by Chained Equations [40]. An imputation model was built, including variables predicting the outcomes, variables that are related to the “missingness” of data, and furthermore, all available midpoint and follow-up effect measure values [40]. Ten complete data sets were created in order for the loss-of-efficiency to be below 5% [41]. All of the imputed datasets were analysed separately as specified below. Rubin’s rules were used to subsequently calculate Pooled estimates [41].

#### Clinical effectiveness

The clinical effectiveness analyses contained two parts. First, linear regression analyses were used to investigate the effectiveness of the TTCM in terms of HR-QOL (generic- and disease-specific), pain, functional status, patient satisfaction and perceived recovery compared with regular care at 3-, 6- and 9-months follow-up. For this purpose, three clusters of control patients (i.e. 3-month cluster, 6-month cluster, and 9-month cluster) were compared with the patients in the intervention group at the corresponding time points. Second, the recovery patterns of the intervention group patients for generic- and disease-specific HR-QOL, pain and functional status during the 9-month follow-up period was studied using GLM for repeated measures [42]. All analyses were adjusted for confounders if necessary (e.g. gender, fracture region, length of stay). Confounding was examined by adding the potential confounding variable to the crude models. If the regression coefficient changed by 10% or more, confounding was considered to be present. Analyses were performed in SPSS Version 22, using a level of significance of *p* < 0.05.

## Results

### Study participants

**Fig. 2:**
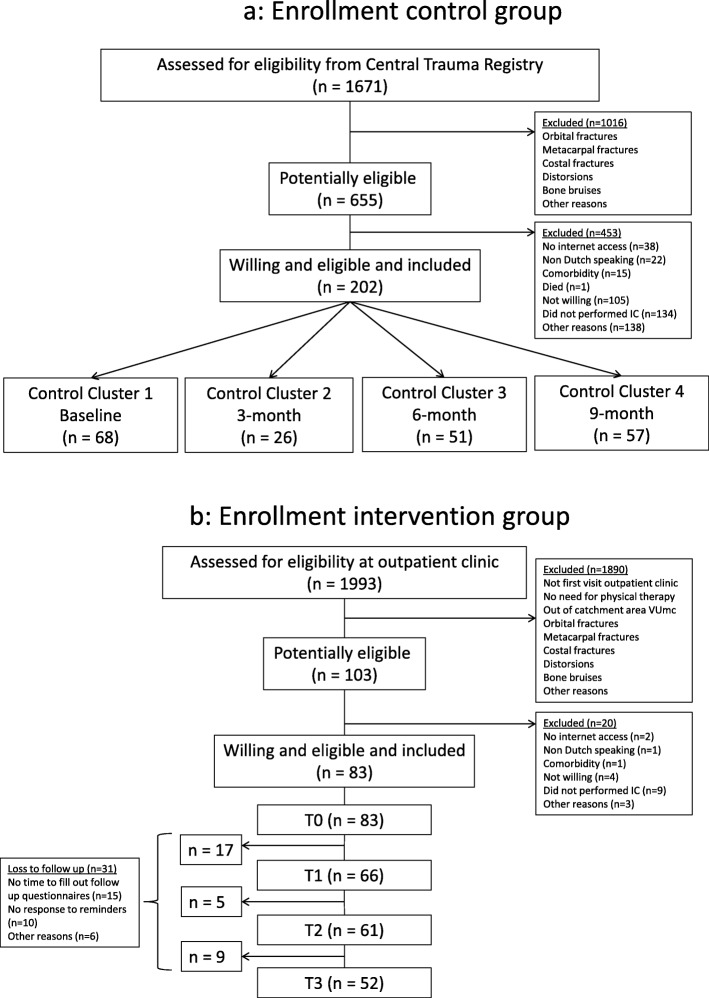
Enrollment. **a** Enrollment of control group participants. **b** Enrollment of intervention group participants

A total of 655 trauma patients were identified as being potentially eligible for participation in the control group. Of them, 453 patients were excluded for various reasons (e.g. did not provide informed consent (*n* = 134), not willing to participate (*n* = 105)). The remaining 202 patients were included in the control group, of which 68 in the baseline cluster, 26 in the 3-month cluster, 51 in the 6-month cluster, and 57 in the 9-month cluster (Fig. 2a). For the intervention group, a total of 103 potentially eligible patients were identified, of whom 20 were eventually excluded for several reasons (e.g. did not provide informed consent (*n* = 9), did not have internet access (*n* = 2)). The remaining 83 patients were included as participants in the intervention group (Fig. 2b).

Baseline characteristics of participants in the four control group clusters and the intervention group are described in Table 2. The majority of these characteristics were similar among participants. However, participants in the intervention group were younger, were more frequently admitted to the hospital, and had lower extremity fractures more often than their control group counterparts.

**Table 2:** Baseline characteristics (patient- and trauma related)

Characteristics	Intervention group	Control group Cluster 1 (baseline)	Control group Cluster 2 (3-month)	Control group Cluster 3 (6-month)	Control group Cluster 4 (9-month)
	Mean (SD) or frequency (%)	Mean (SD) or frequency (%)	Mean (SD) or frequency (%)	Mean (SD) or frequency (%)	Mean (SD) or frequency (%)
N	83	68	26	51	57
Age	43.4 (15.6)	46.8 (14.3)	57.2 (16.0)	50.0 (17.4)	50.5 (17.9)
Gender (M/F)	39/44 (47/53%)	31/37 (46/54%)	13/13 (50/50%)	22/29 (43/57%)	26/31 (46/54%)
Education level					
Low	7 (8.4%)	12 (18.5%)	2 (8.3%)	5 (10.6%)	6 (11.1%)
Middle	19 (22.9%)	16 (24.6%)	4 (16.7%)	14 (29.8%)	16 (29.6%)
High	57 (68.7%)	37 (56.9%)	18 (75.0%)	28 (59.6%)	32 (59.3%)
Medical history					
None	53 (63.9%)	33 (48.5%)	14 (53.8%)	30 (60.0%)	30 (52.6%)
Chronic	14 (16.9%)	21 (30.9%)	7 (26.9%)	9 (18%)	13 (22.8%)
Musculoskeletal	16 (19.3%)	14 (20.6%)	5 (19.2%)	11 (22%)	14 (24.6%)
Trauma type					
Traffic	44 (53.0%)	26 (38.8%)	9 (34.6%)	15 (29.4%)	25 (43.9%)
Work related	0	3 (4.5%)	3 (11.5%)	2 (3.9%)	2 (3.5%)
Fall	27 (32.5%)	13 (19.4%)	9 (34.6%)	20 (39.2%)	17 (29.8%)
Sports	11 (13.3%)	19 (28.4)	5 (19.2%)	9 (17.6%)	9 (15.8%)
Other	1 (1.2%)	6 (9%)	0	5 (9.8%)	4 (7.0%)
Fracture region					
Upper extremity	31 (37.3%)	33 (48.5%)	14 (53.8%)	25 (49.0%)	25 (43.9%)
Lower extremity	41 (49.4%)	25 (36.8%)	9 (34.6%)	16 (31.4%)	19 (33.0%)
Vertebral	7 (8.4%)	0	1 (3.8%)	2 (3.9%)	1 (1.8%)
Multitrauma	4 (4.8%)	10 (14.7%)	2 (7.7%)	8 (15.7%)	12 (21.1%)
ISS (mean, SD)	7.9 (4.4)	9.1 (6.6)	7.7 (5.6)	8.9 (7.8)	8.6 (6.3)
ISS (min-max)	4–26	4–34	4–24	4–43	4–29
Admission hospital	62 (75%)	44 (65%)	8 (31%)	24 (47%)	29 (51%)
Length of stay (days)	7.1 (6.1)	8.2 (7.3)	8.4 (11.1)	10.8 (8.0)	10.0 (11.4)
Surgery	53 (64%)	36 (53%)	5 (19%)	22 (43%)	21 (37%)
TTO (days)^a^	24.3 (14.3)	15.9 (13.1)	11.5 (15.9)	16.0 (15.8)	14.6 (14.7)

^a^*TTO* Time between Trauma and first Outpatient consultation

### Clinical effects

There were no relevant between-group differences in the dependent variable generic HR-QOL (primary outcome measure) at all measurement points (Table 3). However, the mean between-group difference in the dependent variable disease-specific HR-QOL was statistically significant in favor of the intervention group at 9 months (MD -7.96; 95% CI − 14.17 to − 1.75), but not at 3 and 6 months. Patients in the intervention group had statistically significant less pain at 6 (MD -0.87; 95% CI − 1.44 to − 0.29) and 9 months (MD -0.84; 95% CI − 1.38 to − 0.31) than their control group counterparts, but no difference in pain was found at 3 months. There was also a statistically significant difference in functional status favoring the intervention group at 6 months (MD -16.49; 95% CI − 24.39 to − 8.60) and 9 months (MD -20.68; 95% CI − 29.20 to − 12.16), but not at 3 months. Furthermore, participants in the intervention group were statistically significant more satisfied with their total treatment at 3 months (MD 0.77; 95% CI 0.13 to 1.42), but not at 6 and 9 months. At all of the time points, patients in the intervention group were statistically significant more satisfied with the collaboration between primary and secondary care (3 months = MD 1.61; 95% CI 0.72 to 2.51, 6 months = MD 1.78; 95% CI 1.03 to 2.53, and 9 months = MD 1.20; 95% CI 0.42 to 1.97). However, no statistically significant differences were found at any time point for patient satisfaction regarding the treatment at the outpatient clinic (Table 3).

**Table 3:** Treatment effects for primary and secondary outcomes

Outcomes	Intervention group Mean (SD)	Control group Mean (SD)	Treatment effect (crude) MD (95% CI)	Treatment effect (adjusted) MD (95% CI)	Adjusted for^a^
Primary outcome					
HR-QOL (EQ-5D-3L)					
Baseline	0.65 (0.21)	0.70 (0.19)	− 0.05 (− 0.11–0.021)	− 0.031 (− 0.1–0.038)	age, TTO^b^, admission hospital, surgery
3 months	0.78 (0.16)	0.85 (0.13)	− 0.07 (− 0.14–0.006)	− 0.083 (− 0.17–0.001)	age, TTO, fracture region
6 months	0.82 (0.13)	0.81 (0.19)	0.013 (− 0.05–0.08)	0.051 (− 0.02–0.12)	gender, trauma type, TTO, admission hospital, surgery
9 months	0.85 (0.13)	0.81 (0.19)	0.04 (− 0.02–0.10)	0.055 (− 0.01–0.12)	age, medical history, TTO
Secondary outcomes					
Disease-specific HR-QOL (DSQOL-OA)					
Baseline	55.60 (21.35)	50.61 (22.26)	4.99 (−1.88–11.86)	3.65 (− 3.37–10.67)	age, medical history, TTO, fracture region, admission hospital, surgery
3 months	29.56 (20.06)	26.16 (23.35)	3.44 (−5.21–12.09)	0.36 (−8.85–9.58)	age, medical history, fracture region, admission hospital, surgery, length of stay
6 months	22.66 (20.01)	20.21 (20.09)	2.45 (−3.91–8.82)	−3.65 (− 10.38–3.08)	age, medical history, trauma type, TTO, fracture region, ISS, admission hospital, surgery
9 months	17.63 (15.74)	20.45 (21.00)	−2.82 (− 8.41–2.77)	−7.96 (− 14.17 - -1.75)	age, medical history, TTO, fracture region, admission hospital, surgery
Pain (NPRS)					
Baseline	2.84 (1.85)	2.83 (1.93)	0.013 (− 0.60–0.62)	0.34 (− 0.36–1.04)	gender, age, education, medical history, trauma type, TTO, fracture region, ISS, admission hospital, surgery
3 months	1.95 (1.26)	2.54 (1.64)	−0.59 (− 1.2–0.02)	−0.38 (− 1.07–0.32)	TTO, admission hospital, surgery
6 months	1.63 (1.33)	2.31 (2.09)	− 0.68 (− 1.26 - -0.11)	−0.87 (− 1.44 - -0.29)	trauma type
9 months	1.34 (1.11)	2.19 (2.01)	−0.84 (− 1.38 - -0.31)	− 0.84 (− 1.38 - -0.31)	none
Functional status (PSFS)					
Baseline	65.9 (28.89)	58.15 (37.1)	6.84 (−3.69–17.37)	5.90 (− 5.45–17.24)	education, medical history, fracture region
3 months	26.71 (26.82)	34.00 (35.72)	−7.29 (−19.32–4.74)	− 2.02 (− 15.82–11.76)	medical history, trauma type, TTO, fracture region, admission hospital, length of stay
6 months	18.49 (22.06)	29.64 (25.43)	−11.15 (− 18.66 - -3.64)	− 16.49 (− 24.39 - -8.60)	trauma type, TTO, admission hospital, surgery
9 months	18.39 (22.36)	34.39 (30.74)	−16.00 (− 24.09 - -7.90)	−20.68 (− 29.20 - -12.16)	TTO, surgery
Patient satisfaction (total treatment)					
Baseline	7.84 (1.48)	7.32 (2.01)	0.53 (− 0.03–1.08)	0.60 (0.036–1.16)	education
3 months	8.10 (0.99)	7.17 (1.43)	0.93 (0.38–1.47)	0.77 (0.13–1.42)	TTO, admission hospital, surgery, length of stay
6 months	8.09 (1.35)	7.44 (2.04)	0.64 (0.07–1.22)	0.53 (− 0.07–1.13)	admission hospital
9 months	8.09 (1.57)	7.77 (1.18)	0.32 (− 0.19–0.82)	0.24 (− 0.29–0.76)	surgery
Patient satisfaction (outpatient clinic)					
Baseline	7.76 (1.57)	7.33 (2.01)	0.43 (− 0.15–1.00)	0.56 (− 0.018–1.15)	education, medical history
3 months	7.73 (1.55)	7.41 (1.66)	0.31 (−0.37–1.00)	0.15 (− 0.64–0.95)	medical history, TTO, fracture region, admission hospital, surgery, length of stay
6 months	7.98 (1.46)	7.50 (1.92)	0.48 (− 0.09–1.05)	0.39 (−0.25–1.03)	trauma type, fracture region, admission hospital, surgery
9 months	8.22 (1.41)	7.71 (1.36)	0.51 (0.03–0.98)	0.44 (− 0.08–0.95)	age, TTO, surgery
Patient satisfaction (collaboration)					
Baseline	7.16 (2.00)	5.77 (2.57)	1.39 (0.39–2.39)	1.25 (0.24–2.25)	trauma type
3 months	7.40 (1.31)	5.61 (2.19)	1.79 (0.93–2.66)	1.61 (0.72–2.51)	TTO
6 months	7.65 (1.53)	5.86 (2.48)	1.78 (1.03–2.53)	1.78 (1.03–2.53)	none
9 months	7.51 (2.03)	6.13 (2.35)	1.39 (0.65–2.12)	1.20 (0.42–1.97)	admission hospital, surgery

^a^The baseline characteristics mentioned in this column were confounders (changed the regression coefficient with 10% or more)

^b^*TTO* Time between Trauma and first Outpatient consultation (days)

Based on the Global Perceived Effect, 74.6, 78.3, and 84.6% of the intervention group patients were “completely recovered” or “much improved” at 3, 6, and 9 months, respectively. Of the control group patients, 53.8, 53.3 and 75% were “completely recovered” or “much improved” at these time points. At 6 months this effect was statistically significant (OR 3.35; 95% CI 1.32 to 8.49) (Table 4).

**Table 4:** Treatment effect for global perceived effect (“completely recovered” or “much improved”)

GPE Succes	Succes% Intervention group	Succes% Control group	Treatment effect (crude) OR (95% CI)	Treatment effect (adjusted) OR (95% CI)	Adjusted for^a^
3 months	74.6	53.8	2.37 (0.85–6.62)	2.39 (0.69–8.20)	age, fracture region, length of stay
6 months	78.3	53.3	2.99 (1.24–7.23)	3.35 (1.32–8.49)	fracture region
9 months	84.6	75.0	1.16 (0.46–2.95)	1.21 (0.45–3.28)	medical history, TTO^b^

^a^The baseline characteristics mentioned in this column were confounders (changed the regression coefficient with 10% or more)

^b^*TTO* Time between Trauma and first Outpatient consultation (days)

### Recovery pattern of patients in the intervention group

**Fig. 3:**
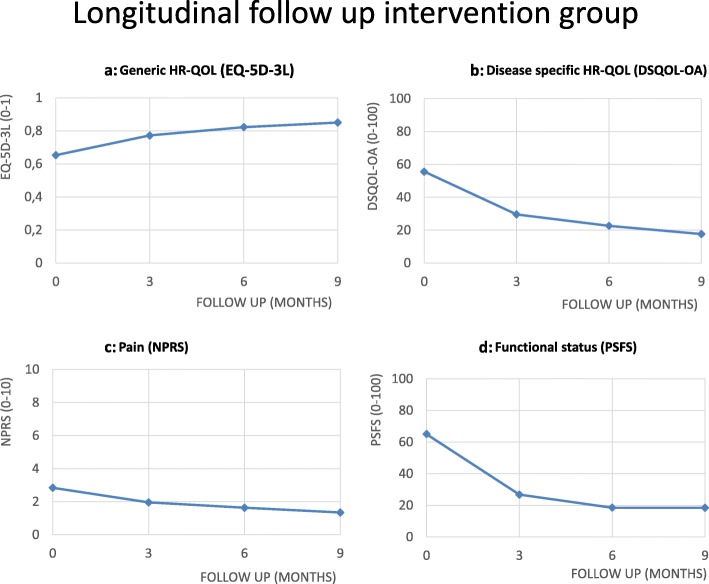
Longitudinal follow up intervention group. **a** Generic HR-QOL (EQ-5D-3L). **b** Disease specific HR-QOL (DSQOL-OA). **c** Pain (NPRS). **d** Functional status (PSFS)

During the nine-month follow-up period, generic HR-QOL (F = 18.43; *p* = 0.000), disease-specific HR-QOL (F = 6.18; *p* = 0.001), pain (F = 17.16; *p* = 0.000), and functional status (F = 65.05; *p* = 0.000) statistically significantly improved in a linear fashion among intervention group patients (Fig. 3a, b, c and d).

## Discussion

Traumatic injury poses a substantial economic burden to society. However, little is currently known about how to optimally organize the post-clinical rehabilitation process of trauma patients. Therefore, the current study developed and evaluated the TTCM, the first transmural care model for the rehabilitation of trauma patients in primary care [9, 10, 23].

### Important study findings and comparison with the literature

Our results indicate that the TTCM statistically significantly improved disease-specific HR-QOL, pain, functional status, patient satisfaction and perceived recovery among mild, moderate and severe trauma patients. It is important to mention, however, that even though no statistically significant effects were found for generic HR-QOL, the identified mean difference can be regarded as clinically relevant at 6 months (MD 0.051; 95% CI − 0.02 to 0.12) and 9 months (MD 0.055; 95% CI − 0.01 to 0.12). To illustrate, estimates of the minimal clinical important difference (MCID) for the EQ-5D range from 0.03 among patients with low back pain [43] to 0.52 in patients with recurrent lumbar stenosis [44]. In light of this finding it is also important to bear in mind that the current study was not powered to detect a clinically meaningful difference in generic HR-QOL due to its explorative nature.

### Strengths and limitations

The present study population covers a broad range in trauma patients, with an ISS ranging from 4 to 43. This is an important strength, as the majority of studies only included major trauma patients with an ISS > 16 [4, 14, 45]. Since our study population includes mild, moderate and severely injured patients, the TTCM is likely to be effective in the entire group of trauma patients. However, future research is necessary to examine whether certain subgroups of trauma patients respond in different ways to the TTCM than others.

A second important strength of this study is its clinical relevance as well as the fact that it was the first to develop and evaluate a transmural care model for the post-clinical rehabilitation of trauma patients. Other strengths include its use of a broad spectrum of measurement instruments, covering all domains of the International Classification of Functioning, Disability and Health (ICF) [46], its use of validated questionnaires, as well as its pragmatic design (i.e. daily practice was resembled as much as possible).

The study also had some limitations. Even though the applied modified controlled-before-and-after design was regarded as the most optimal research design within the available resources, it is susceptible to many kinds of bias. Examples of such kinds of bias are selection bias, recall bias, regression to the mean, the Hawthorne effect, and repeat testing bias. Of them, selection bias is probably most likely, meaning that the study groups have a different composition regarding various etiological factors. A multicenter randomized controlled trial would therefore be the next step in order to study the TTCM’s effectiveness more robustly. In spite of the fact the all participating trauma patients met the same inclusion criteria, we observed some baseline differences in age (intervention group patients were younger) and admission to hospital (75% of intervention group patients were admitted to the hospital, compared to 51% in control group). Based on the recommendations of de Boer et al. we decided not to statistically test baseline differences across study groups [47]. They postulate that statistically testing of baseline differences ignores the fact that the prognostic strength of a variable is also important when the interest is in e.g. adjustment for confounding. On top of that, our study was not powered to detect relevant differences at baseline, so possibly relevant differences may also turn out not to be statistically significant. Nonetheless, if we found the addition of a certain baseline variable to change the regression coefficient by more than 10%, they were added to the final models. Another potential limitation was the absence of a sample size calculation. We based the sample size on our estimate of the number of patients that could potentially be included within the time frame and financial constraints of this study. A sample size calculation is preferable in future research, to make sure that the study is not underpowered to detect clinically meaningful effect differences. Another limitation is the fact that we were not able to identify what components of the TTCM were responsible for the positive effects. To illustrate, the better functional outcomes could be the result of an improved communication strategy between the multidisciplinary hospital team and the primary care physical therapist. On the other hand, the better outcomes may have been the result of a better educated and more experienced network of primary care physical therapists. It would be interesting to identify the critical ingredient of this relatively complex intervention. One might also argue, however, that the sum is greater than the individual parts and therefore there is probably no such thing as a critical ingredient. Future research can possibly provide more insight into whether separate TTCM components are accountable for specific effects.

## Conclusions

This study provides preliminary evidence that the TTCM is effective in improving patient-related outcome measures, such as disease-specific HR-QOL and functional status. A multicenter, and ideally randomized controlled trial, is required to confirm these results.

## Data Availability

The datasets generated and analysed during the current study are not publicly available due to the volume, but are available from the corresponding author on reasonable request.

## Abbreviations

DALY: Disability-Adjusted Life Years
DSQOL-OA: Overall disease-specific HR-QOL
GARS: Groningen Activity Restriction Scale
GPE: Global Perceived Effect
HR-QOL: Health-Related Quality Of Life
ICF: International Classification of Functioning, Disability and Health
ISS: Injury Severity Score
LEFS: Lower Extremity Functional Scale
MCID: Minimal Clinical Important Difference
NRS: Numeric Rating Scale
NTR: The Netherlands National Trial Register
PSFS: Patient Specific Function Scale
RMDS: Roland Morris Disability Score
TTCM: Transmural Trauma Care Model
TTO: Time between Trauma and first Outpatient consultation
VUmc: Amsterdam UMC, Vrije Universiteit Amsterdam

## References

[1] World Health Organisation (2002). The world health report 2002 - reducing risks, promoting healthy life.

[2] Lozano R, Naghavi M, Foreman K, Lim S, Shibuya K, Aboyans V, Abraham J, Adair T, Aggarwal R, Ahn SY (2012). Global and regional mortality from 235 causes of death for 20 age groups in 1990 and 2010: a systematic analysis for the global burden of disease study 2010. Lancet.

[3] Murray CJ, Vos T, Lozano R, Naghavi M, Flaxman AD, Michaud C, Ezzati M, Shibuya K, Salomon JA, Abdalla S (2012). Disability-adjusted life years (DALYs) for 291 diseases and injuries in 21 regions, 1990-2010: a systematic analysis for the global burden of disease study 2010. Lancet.

[4] Holbrook TL, Anderson JP, Sieber WJ, Browner D, Hoyt DB (1999). Outcome after major trauma: 12-month and 18-month follow-up results from the trauma recovery project. J Trauma.

[5] Stalp M, Koch C, Ruchholtz S, Regel G, Panzica M, Krettek C, Pape HC (2002). Standardized outcome evaluation after blunt multiple injuries by scoring systems: a clinical follow-up investigation 2 years after injury. J Trauma.

[6] van der Sluis CK, Eisma WH, Groothoff JW, Ten Duis HJ (1998). Long-term physical, psychological and social consequences of a fracture of the ankle. Injury.

[7] Siebenga J, Segers MJ, Leferink VJ, Elzinga MJ, Bakker FC, Duis HJ, Rommens PM, Patka P (2007). Cost-effectiveness of the treatment of traumatic thoracolumbar spine fractures: nonsurgical or surgical therapy?. Indian J Orthop.

[8] Fakhry SM, Martin B, Al Harakeh H, Norcross ED, Ferguson PL (2013). Proportional costs in trauma and acute care surgery patients: dominant role of intensive care unit costs. J Am Coll Surg.

[9] Lansink KW, Leenen LP (2007). Do designated trauma systems improve outcome?. Curr Opin Crit Care.

[10] MacKenzie EJ, Rivara FP, Jurkovich GJ, Nathens AB, Frey KP, Egleston BL, Salkever DS, Scharfstein DO (2006). A national evaluation of the effect of trauma-center care on mortality. N Engl J Med.

[11] Haas B, Jurkovich GJ, Wang J, Rivara FP, Mackenzie EJ, Nathens AB (2009). Survival advantage in trauma centers: expeditious intervention or experience?. J Am Coll Surg.

[12] Celso B, Tepas J, Langland-Orban B, Pracht E, Papa L, Lottenberg L, Flint L (2006). A systematic review and meta-analysis comparing outcome of severely injured patients treated in trauma centers following the establishment of trauma systems. J Trauma.

[13] Ardolino A, Sleat G, Willett K (2012). Outcome measurements in major trauma-results of a consensus meeting. Injury.

[14] Gabbe BJ, Sutherland AM, Hart MJ, Cameron PA (2010). Population-based capture of long-term functional and quality of life outcomes after major trauma: the experiences of the Victorian state trauma registry. J Trauma.

[15] Innocenti F, Del Taglia B, Coppa A, Trausi F, Conti A, Zanobetti M, Pini R (2015). Quality of life after mild to moderate trauma. Injury.

[16] Toien K, Bredal IS, Skogstad L, Myhren H, Ekeberg O (2011). Health related quality of life in trauma patients. Data from a one-year follow up study compared with the general population. Scand J Trauma Resusc Emerg Med.

[17] Mock C, MacKenzie E, Jurkovich G, Burgess A, Cushing B, deLateur B, McAndrew M, Morris J, Swiontkowski M (2000). Determinants of disability after lower extremity fracture. J Trauma.

[18] Kempen GI, Scaf-Klomp W, Ranchor AV, Sanderman R, Ormel J (2001). Social predictors of recovery in late middle-aged and older persons after injury to the extremities: a prospective study. J Gerontol B Psychol Sci Soc Sci.

[19] Franche RL, Krause N (2002). Readiness for return to work following injury or illness: conceptualizing the interpersonal impact of health care, workplace, and insurance factors. J Occup Rehabil.

[20] Munneke M, Nijkrake MJ, Keus SH, Kwakkel G, Berendse HW, Roos RA, Borm GF, Adang EM, Overeem S, Bloem BR, ParkinsonNet Trial Study G (2010). Efficacy of community-based physiotherapy networks for patients with Parkinson’s disease: a cluster-randomised trial. Lancet Neurol.

[21] Voorn VM, Vermeulen HM, Nelissen RG, Kloppenburg M, Huizinga TW, Leijerzapf NA, Kroon HM, Vliet Vlieland TP, van der Linden HM (2013). An innovative care model coordinated by a physical therapist and nurse practitioner for osteoarthritis of the hip and knee in specialist care: a prospective study. Rheumatol Int.

[22] de Rooij M, van der Leeden M, Cheung J, van der Esch M, Hakkinen A, Haverkamp D, Roorda LD, Twisk J, Vollebregt J, Lems WF, Dekker J (2016). Efficacy of tailored exercise therapy on physical functioning in patients with knee osteoarthritis and comorbidity: a randomized controlled trial. Arthritis Care Res (Hoboken).

[23] Wiertsema SH, van Dongen JM, Geleijn E, Schothorst M, Bloemers FW, de Groot V, Ostelo RW (2017). Evaluation of a new Transmural trauma care model (TTCM) for the rehabilitation of trauma patients: a study protocol. BMC Health Serv Res.

[24] Wiertsema SH, van Dongen JM, Geleijn E, Huijsmans RJ, Bloemers FW, de Groot V, Ostelo RW (2019). Cost-effectiveness of the Transmural trauma care model (TTCM) for the rehabilitation of trauma patients. Int J Technol Assess Health Care.

[25] Higgins JTP, Green S. Cochrane handbook for systematic reviews of interventions. Version 5.1.0 [Updated March 2011]. The Cochrane Collaboration, 2011. Available from https://www.cochrane-handbook.org.

[26] Netwerk Traumarevalidatie VUmc [http://traumarevalidatie.nl/].

[27] Lamers L, Stalmeier P, McDonnell J, Krabbe P, van Busschbach J (2005). Kwaliteit van leven meten in economische evaluaties: het Nederlandse EQ-5D-tarief. Ned Tijdschr Geneeskd.

[28] Hudak PL, Amadio PC, Bombardier C (1996). Development of an upper extremity outcome measure: the DASH (disabilities of the arm, shoulder and hand) [corrected]. The upper extremity collaborative group (UECG). Am J Ind Med.

[29] Veehof MM, Sleegers EJ, van Veldhoven NH, Schuurman AH, van Meeteren NL (2002). Psychometric qualities of the Dutch language version of the disabilities of the arm, shoulder, and hand questionnaire (DASH-DLV). J Hand Ther.

[30] Binkley JM, Stratford PW, Lott SA, Riddle DL (1999). The Lower Extremity Functional Scale (LEFS): scale development, measurement properties, and clinical application. North American Orthopaedic rehabilitation research network. Phys Ther.

[31] Hoogeboom TJ, de Bie RA, den Broeder AA, van den Ende CH (2012). The Dutch lower extremity functional scale was highly reliable, valid and responsive in individuals with hip/knee osteoarthritis: a validation study. BMC Musculoskelet Disord.

[32] Roland M, Morris R (1983). A study of the natural history of back pain. Part I: development of a reliable and sensitive measure of disability in low-back pain. Spine (Phila Pa 1976).

[33] Brouwer S, Kuijer W, Dijkstra PU, Goeken LN, Groothoff JW, Geertzen JH (2004). Reliability and stability of the Roland Morris disability questionnaire: intra class correlation and limits of agreement. Disabil Rehabil.

[34] Kempen GI, Miedema I, Ormel J, Molenaar W (1996). The assessment of disability with the Groningen Activity Restriction Scale. Conceptual framework and psychometric properties. Soc Sci Med.

[35] Jansen L, Steultjens MP, Holtslag HR, Kwakkel G, Dekker J (2010). Psychometric properties of questionnaires evaluating health-related quality of life and functional status in polytrauma patients with lower extremity injury. J Trauma Manag Outcomes.

[36] Von KM, Jensen MP, Karoly P (2000). Assessing global pain severity by self-report in clinical and health services research. Spine (Phila Pa 1976).

[37] Chatman AB, Hyams SP, Neel JM, Binkley JM, Stratford PW, Schomberg A, Stabler M (1997). The patient-specific functional scale: measurement properties in patients with knee dysfunction. Phys Ther.

[38] Beurskens AJ, de Vet HC, Koke AJ, Lindeman E, van der Heijden GJ, Regtop W, Knipschild PG (1999). A patient-specific approach for measuring functional status in low back pain. J Manip Physiol Ther.

[39] Kamper SJ, Ostelo RW, Knol DL, Maher CG, de Vet HC, Hancock MJ (2010). Global perceived effect scales provided reliable assessments of health transition in people with musculoskeletal disorders, but ratings are strongly influenced by current status. J Clin Epidemiol.

[40] Azur MJ, Stuart EA, Frangakis C, Leaf PJ (2011). Multiple imputation by chained equations: what is it and how does it work?. Int J Methods Psychiatr Res.

[41] White IR, Royston P, Wood AM (2011). Multiple imputation using chained equations: issues and guidance for practice. Stat Med.

[42] Twisk JWR (2013). Applied longitudinal data analysis for epidemiology. A practical guide.

[43] Soer R, Reneman MF, Speijer BL, Coppes MH, Vroomen PC (2012). Clinimetric properties of the EuroQol-5D in patients with chronic low back pain. Spine J.

[44] Parker SL, Mendenhall SK, Shau DN, Adogwa O, Anderson WN, Devin CJ, McGirt MJ (2012). Minimum clinically important difference in pain, disability, and quality of life after neural decompression and fusion for same-level recurrent lumbar stenosis: understanding clinical versus statistical significance. J Neurosurg Spine.

[45] Holtslag HR, Post MW, Lindeman E, Van der Werken C (2007). Long-term functional health status of severely injured patients. Injury.

[46] World Health Organisation (2001). International classification of functioning, disability and health (ICF).

[47] de Boer MR, Waterlander WE, Kuijper LD, Steenhuis IH, Twisk JW (2015). Testing for baseline differences in randomized controlled trials: an unhealthy research behavior that is hard to eradicate. Int J Behav Nutr Phys Act.

